# Laparoscopic distal gastrectomy skill evaluation from video: a new artificial intelligence-based instrument identification system

**DOI:** 10.1038/s41598-024-63388-y

**Published:** 2024-05-30

**Authors:** Shiro Matsumoto, Hiroshi Kawahira, Kyohei Fukata, Yasunori Doi, Nao Kobayashi, Yoshinori Hosoya, Naohiro Sata

**Affiliations:** 1 Department of Surgery, Division of Gastroenterological, General and Transplant Surgery, Jichi Medical University, Tochigi, Japan; 2 Medical Simulation Center, Jichi Medical University, Tochigi, Japan; 3 Anaut Co., Ltd., Tokyo, Japan

**Keywords:** Gastroenterology, Medical research

## Abstract

The advent of Artificial Intelligence (AI)-based object detection technology has made identification of position coordinates of surgical instruments from videos possible. This study aimed to find kinematic differences by surgical skill level. An AI algorithm was developed to identify X and Y coordinates of surgical instrument tips accurately from video. Kinematic analysis including fluctuation analysis was performed on 18 laparoscopic distal gastrectomy videos from three expert and three novice surgeons (3 videos/surgeon, 11.6 h, 1,254,010 frames). Analysis showed the expert surgeon cohort moved more efficiently and regularly, with significantly less operation time and total travel distance. Instrument tip movement did not differ in velocity, acceleration, or jerk between skill levels. The evaluation index of fluctuation β was significantly higher in experts. ROC curve cutoff value at 1.4 determined sensitivity and specificity of 77.8% for experts and novices. Despite the small sample, this study suggests AI-based object detection with fluctuation analysis is promising because skill evaluation can be calculated in real time with potential for peri-operational evaluation.

## Introduction

Laparoscopic surgery is widely performed due to its minimally invasive nature and precise manipulation^1^, but is more difficult than open surgery and requires specialized training^2^. Various methods have been developed for accurate and objective skill evaluation^3^. The Japan Society of Endoscopic Surgery established the Endoscopic Surgical Skill Qualification System^4^, and surgeries performed by qualified surgeons have been proven to have fewer complications^5^. The Global Operative Assessment of Laparoscopic Skills (GOALS) was developed in Canada as an evaluation rubric and has proven useful for skill evaluation^6,7^. In these skill evaluation systems, experts evaluate unedited videos according to a score sheet, indicating that surgical skills can be evaluated solely from surgical videos. Although the Endoscopic Surgical Skill Qualification System and GOALS are considered reliable, reliability depends on the long-term experience of the evaluator^8,9^. These evaluation methods also require evaluators to spend considerable effort and time reviewing the entire surgical video. Furthermore, this method is not “real time” as it relies on expert evaluation of videos from past procedures^10^. If a real-time evaluation method is developed, surgeons may be able to receive feedback on their evaluations and potentially improve peri-operationally. Real-time evaluation may also ensure medical safety through notifications of deviations from surgical plans.

In studies of skill evaluation in the laparoscopic training box, kinematic data were obtained by sensors attached to the tips of surgical instruments or trocars to evaluate surgical skill^11,12^. However, these methods in clinical surgery are problematic due to sterility and performance issues^13,14^. Therefore, kinematic data on surgical instruments in clinical surgery are largely unknown.

In recent years, laparoscopic surgery videos increasingly have been analyzed using Artificial Intelligence (AI), including instrument recognition and detection, phase recognition, anatomy recognition and detection, action recognition, surgery time prediction, and gauze recognition^15^. Object detection technology has made identifying the position of the surgical instrument tip possible from surgical video. This method resolves problems of sterility and impact on performance. Additionally, extensive video observation is not required, and quantitative evaluations may be performed in real time. To date, only a few studies have used AI to identify coordinates of the instrument tip position to evaluate surgical skill, and these studies are limited to heat-map assessments of tip position and total travel distance^16,17^. Detailed studies of kinematic differences by skill level do not yet exist.

The present study aimed to find kinematic differences by skill level. To do this, an AI algorithm was developed that can accurately identify the coordinates of the surgical instrument tip in distal gastrectomy videos.

## Materials and methods

### Participants

This study was conducted under the Declaration of Helsinki and was approved by the Institutional Review Board of Jichi Medical University (No.23–043) in Japan. This study analyzed videos of previous surgeries, and the right to opt-out refusal was secured from the patients. Written informed consent was obtained from surgeons subject to skill evaluation. Six gastric surgeons from the Department of Surgery, Division of Gastroenterological, General and Transplant Surgery, Jichi Medical University participated in the study: three were experts who had performed more than 100 laparoscopic gastrectomies, and three were novices who had performed less than 20 laparoscopic gastrectomies (Table 1).

**Table 1:** Descriptive data of surgeon expert and novice group participants.

	Expert n = 3			Novice n = 3		
Age in years	49	47	44	40	37	37
Gender	M	M	M	M	M	M
Dominant hand	R	R	R	R	R	R
Years of laparoscopic surgery experience	23	22	20	14	13	12
No. of laparoscopic gastrectomy surgical cases	138	181	110	19	7	5

### Videos for analysis

All laparoscopic distal gastrectomy videos were taken between 2019 and 2022, and three from each surgeon for a total of 18 videos were evaluated. Expert group members selected videos of their three most recent cases during the study period, and novice group members selected videos of the first three career cases. The videos were recorded at 30fps and in HD quality using a 3D curved tip video scope (LTF-S300-10-3D; Olympus, Tokyo, Japan) and 3D video processor (VISERA ELITE II OTV-S300; Olympus, Tokyo, Japan). Because surgeons from a single institution participated, the surgical procedure was standardized and constant: Dissection of the greater omentum segment, takedown of the transverse mesocolon, subpyloric lymph node dissection, right gastroepiploic vascular dissection, and vascular dissection of the greater curvature side of the duodenum.

### AI development

**Figure 1:**
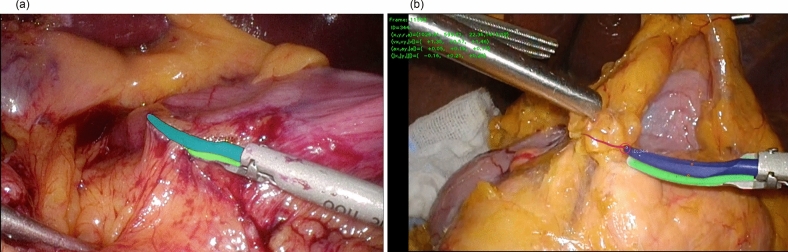
(**a**) Annotated image. The active blade tip, active blade, and tissue pad of the HARMONIC shears were annotated. (**b**) Screen shot of AI surgical video analysis showing coloring of HARMONIC shears. The active blade was colored blue, the tissue pad was colored green, and the tip of the active blade was surrounded by a purple circle and colored to leave an afterimage of the previous 10 frames. The X and Y coordinates, velocity, acceleration, and jerk of the tip are displayed at the top left of the screen.

Analysis was performed using a unique AI algorithm developed by Anaut Co., Ltd. (Tokyo) based on DeepLab v2 developed by Google LLC. As the training data set, 1,080 still images were culled from surgical videos of 6 cases other than the 18 cases to be evaluated. A pair of ultrasonic coagulation shears (HARMONIC HD1000i, Johnson & Johnson, New Jersey, USA) was used in all surgeries, and models were trained by annotating the active blade tip, active blade, and tissue pad of the HARMONIC shears (Fig. 1a) and analyzed by the learned models.

### Video analysis

In the AI analysis of the surgical video, the active blade was colored blue, the tissue pad was colored green, and the tip of the active blade was surrounded by a purple circle and colored to leave an afterimage of the previous 10 frames. The X and Y coordinates, velocity, acceleration, and jerk of the tip are displayed at the top left of the screen (Fig. 1b). Part of the video can be found in Online Resource 1.

### Evaluation accuracy

As the test dataset for accuracy evaluation, each video was divided into 11 parts of equal length, and the second and subsequent top images were extracted. For example, in an 11,000-frame video, ten images at frame count -001 (e.g., 1001, 2001, 3001, etc. to 10,001) were extracted. As ten images were extracted per case, there was a total of 180 images for accuracy evaluation.

Accuracy was evaluated using True Positive Rate (TPR), False Positive Rate (FPR), and Dice. Dice and TPR are the most commonly used indices for machine learning performance evaluation^18^.

### Fluctuation analysis

When the time-series power spectral density S(f) in the following formula has β close to 1, there is said to be a 1/f fluctuation.

$$S(f)\propto \frac{1}{{f}^{\beta }}$$

Taking the logarithm of both sides, the slope of the approximate line plotted on a logarithmic scale is β. β = 0 is said to be white noise and represents a disorderly situation. β = 1 is a 1/f fluctuation, and as β approaches 2, the movement is regular^19^.

$$logS(f)\propto log(\frac{1}{{f}^{\beta }})=-\beta logf$$

We calculated movement characteristics by frequency analysis of data arranged in a time series of travel distances. Using Python Ver. 3.9, time series data were separated every 3 s, power spectra were calculated by fast Fourier transform, and plotted on a logarithmic scale. Fluctuations were analyzed by drawing an approximate straight line using the least squares method and evaluating its slope β.

### Code availability

Part of the data and the code for calculating slope β can be found in Online Resource 2,3.

### Statistical analysis

The total number of frames and the number of frames with the surgical shears in view were calculated. The X and Y coordinates on screen of the shear tips were calculated from the video, followed by the kinematic indices of distance traveled, velocity, acceleration, jerk, and the fluctuation index β. R (The R Foundation for Statistical Computing, Vienna, Austria, version 4.1.0) was used for statistical evaluation. Statistical post hoc power was calculated using G*Power 3.1.9.7^20^.

A density plot was used to evaluate the distance traveled per second. This plot expresses the shape of the data distribution as a curve based on kernel density estimation and expresses the distribution more smoothly than a histogram. The area between the curve and the x-axis was equal to 1 for both groups, adjusting for the difference in surgical time between the two groups.

**Figure 2:**
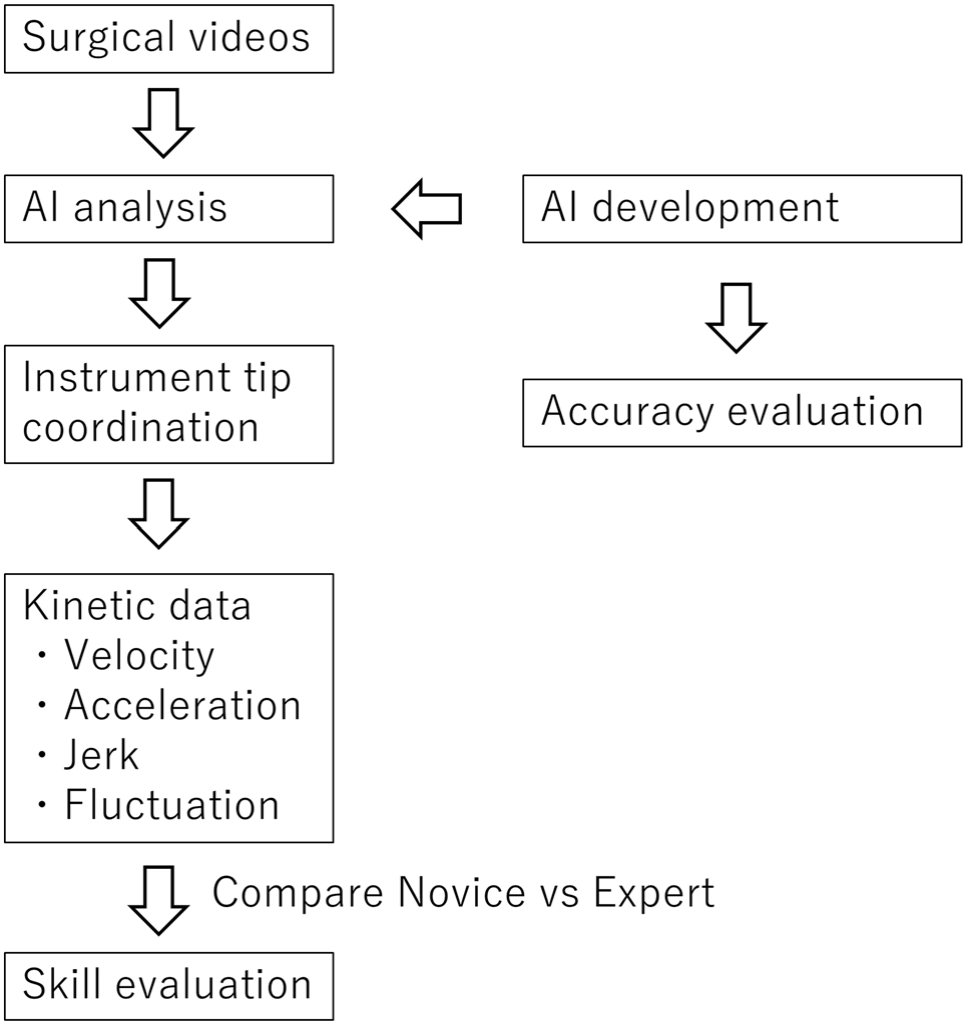
Structure of the study.

The primary outcome of this study was to identify indices that differ according to skill level, and the secondary outcome was to identify cutoff values for those indices. The design of this study is shown in Fig. 2.

## Results

In total, 11.6 h (1,254,010 frames) of videos were extracted from 18 cases and analyzed.

### Accuracy

Of the accuracy evaluation dataset consisting of 180 images, 114 showed the active blade in whole or in part, and 90 of these showed the tip clearly. In the active blade evaluation from 114 images, the TPR was 0.873, the FPR was 0.0041, and the Dice was 0.837. The Active blade tip was evaluated using 90 images, and when inferred within the ground truth tip area, was judged as True. The TPR was 0.813, the FPR was 0.111 and the Dice was 0.846.

### Time

**Figure 3:**
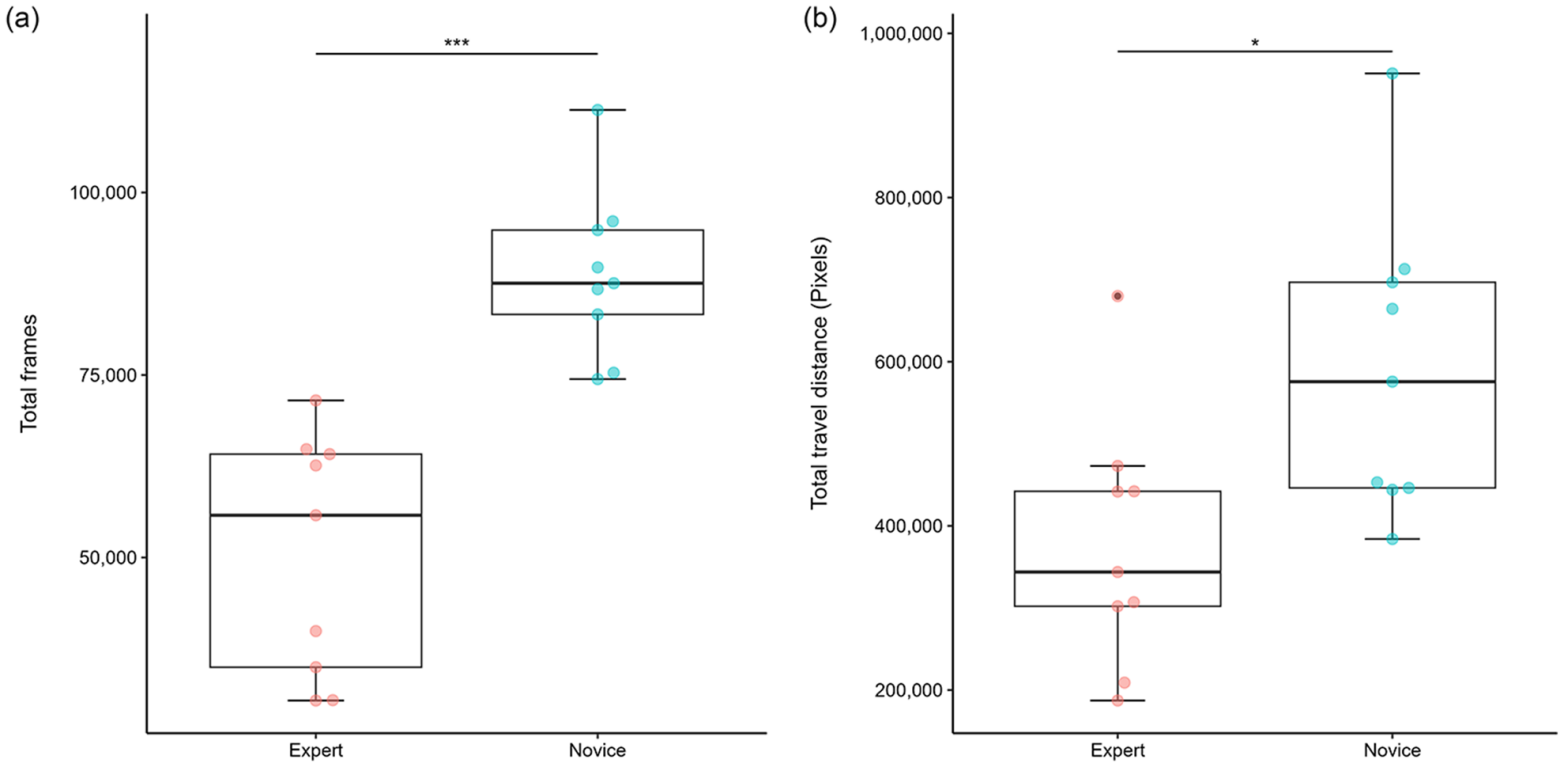
(**a**) Box-plot of between-group time (total frames) differences. The average number of frames per surgery was 50,519 frames (28.1 min) for the Expert group vs. 88,815 frames (49.3 min) for the Novice group, with the Expert group taking significantly less time. (P < 0.001, Mann–Whitney U-test). (**b**) Box-plot of between-group time total travel distance differences. The total travel distance of the tip was 343,794 pixels in the Expert group vs. 575,721 pixels, which was significantly shorter in the Expert group. (P = 0.011, Mann–Whitney U-test).

The average number of frames per surgery was 50,519 frames (28.1 min) for the Expert group vs. 88,815 frames (49.3 min) for the Novice group, with the Expert group taking significantly less time (Fig. 3a, P < 0.001, Mann–Whitney U-test).

The shears occurrence rate (number of frames with the tip of the shears on screen/total frames) was 61.6% in the Expert group vs. 50.4% in the Novice group, and the Expert group tended to spend a higher percentage of time working with the shears, but the difference was not significant (P = 0.063, Mann–Whitney U-test).

### Kinematic indices

**Figure 4:**
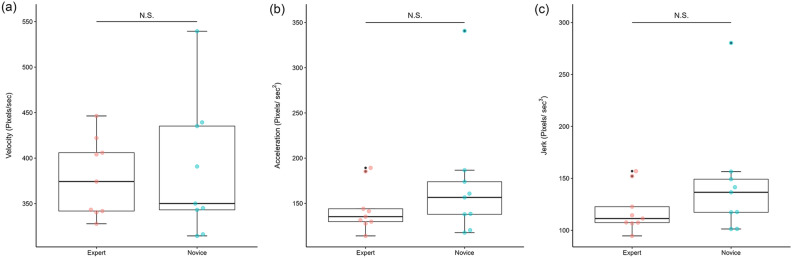
(**a**) Box-plot of between-group velocity differences. The velocity of the tip was 374 pixels/sec for the Expert group vs. 350 for the Novice group. (P = 1.000, Mann–Whitney U-test). (**b**) Box-plot of between-group acceleration differences. The acceleration of the tip was 135 pixels/sec^2^ for the Expert group vs. 156 for the Novice group. (P = 0.436, Mann–Whitney U-test). (**c**) Box-plot of between-group jerk differences. The jerk of the tip was 111 pixels/sec^3^ for the Expert group vs. 136 for the Novice group. (P = 0.340, Mann–Whitney U-test).

**Figure 5:**
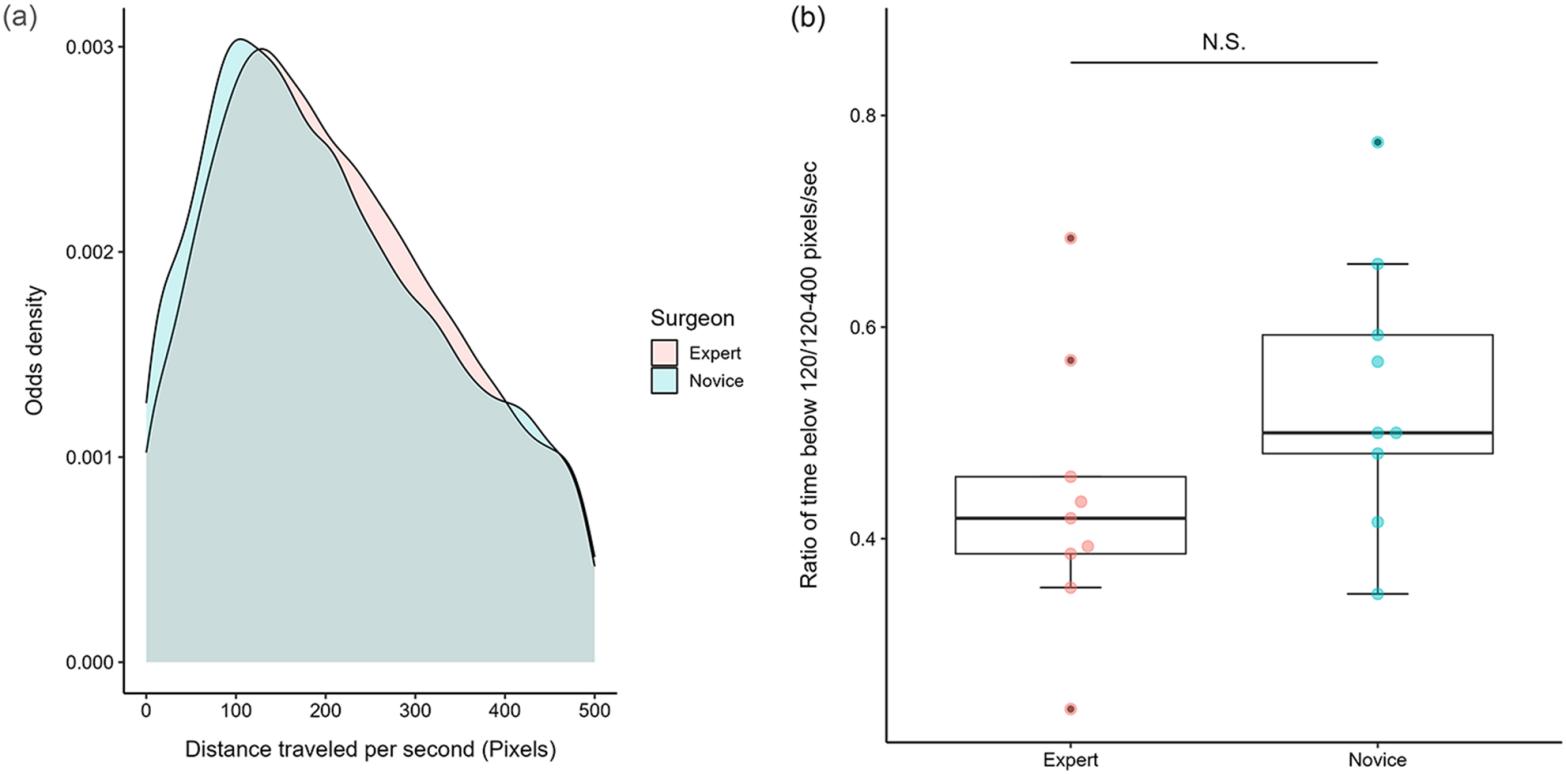
(**a**) Kernel density estimation plot of between-group distance traveled per second differences. The two groups reversed after 120 pixels in the density plot. (**b**) Ratio of time below 120/120–400 pixels/sec 0.419 for the Expert group vs. 0.500 for the Novice group, indicating that the Novice group tended to have a longer time of hovering tip motion with a distance traveled in 1 s below 120 pixels. (P = 0.133, Mann–Whitney U-test).

The total travel distance was 343,794 pixels in the Expert group vs. 575,721 pixels, which was significantly shorter in the Expert group (Fig. 3b,  P = 0.011, Mann–Whitney U-test). There were no significant differences in velocity, acceleration, or jerk at the HARMONIC tip between the two groups (Fig. 4a,b,c). There was also no difference in the average distance traveled per second, but the two groups reversed after 120 pixels in the density plot (Fig. 5a), and the ratio (ratio of time below 120/120–400) was 0.419 for the Expert group vs. 0.500 for the Novice group, indicating that the Novice group tended to have longer hovering tip motion time with a distance traveled in 1 s below 120 pixels (Fig. 5b, P = 0.133, Mann–Whitney U-test).

**Figure 6:**
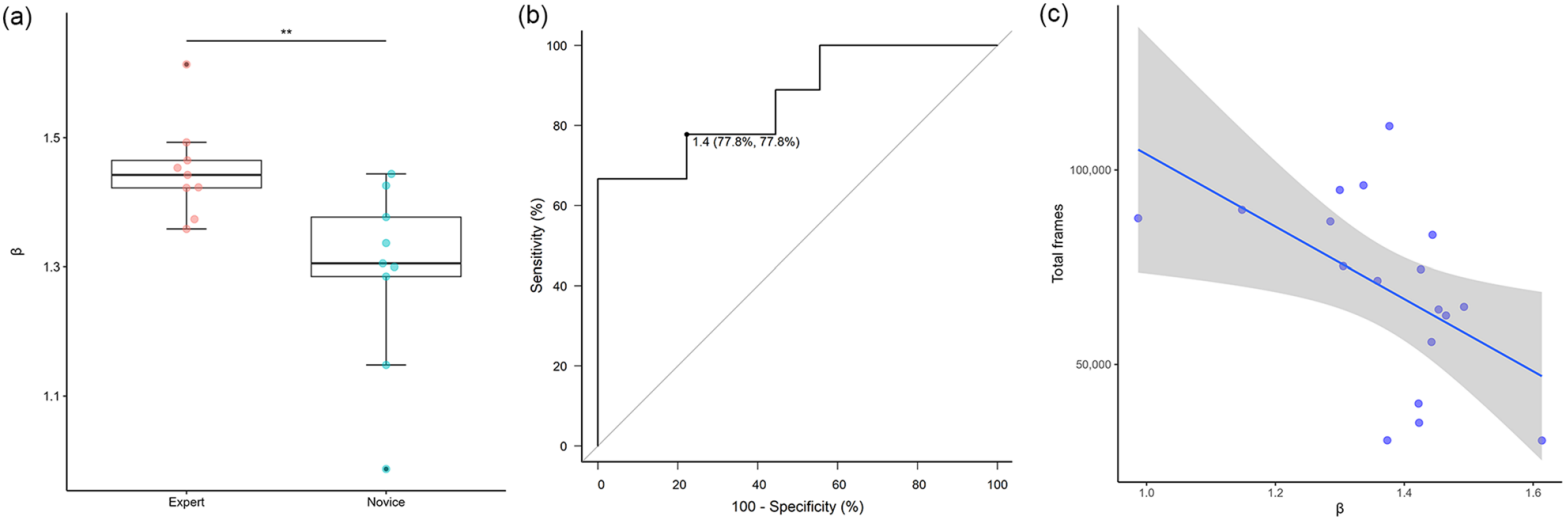
(**a**) Box-plot of between-group β-value fluctuation indicator differences. β was significantly higher for Expert at 1.44 vs. 1.30 for Novice. (P = 0.008, Mann–Whitney U-test) This indicates that the Expert group has more regular movements. (**b**) ROC curve. Using a cutoff value of 1.4, Expert and Novice could be differentiated with both sensitivity and specificity of 77.8%. (**c**) Graph of β-value fluctuation (x-axis) and total frames representing time (y-axis) with line of best fit based on Spearman's rank correlation coefficients (rho = -0.616).

In the fluctuation evaluation, β was significantly higher for Expert at 1.45 vs. 1.29 for Novice (Fig. 6a, P = 0.008, Mann–Whitney U-test). This indicates that the Expert group had more regular movements. ROC curves were drawn and cutoff values for Expert and Novice were calculated; using a cutoff value of 1.4, Expert and Novice could be differentiated with both sensitivity and specificity of 77.8% (Fig. 6b). Spearman's rank correlation coefficient between operating time and β was rho = -0.616, indicating a moderate negative correlation (Fig. 6c).

As this was a single-center study and the available surgical videos were limited, no prior sample size calculations were performed. However, post hoc power calculation for between-sample Mann Whitney U tests of fluctuation β differences showed that our experiment reached 0.81 power for the effect size (Cohen’s d = 1.46) at α = 0.05.

## Discussion

The present study aimed to find kinematic differences by skill level. We developed an AI algorithm that can accurately identify coordinates of the surgical instrument tip in videos of distal gastrectomy. The total number of frames, total travel distance, and the fluctuation index β differed by skill level.

Advances in AI have made surgical instrument detection possible from surgical videos. To apply this technology to surgeon skill evaluation, position information at the pixel level by segmentation is necessary. Therefore, rather than Object Detection which classifies and displays bounding boxes, we used Semantic Segmentation. Importantly, many challenges can hinder object detection accuracy in laparoscopic surgery, such as mist from ultrasonic coagulation shears, blood, and dirt on instruments^10^. In addition, ultrasonic coagulation shears exude mist near the tip, so identification of the tip is difficult compared with other surgical instruments. Prior studies have reported high accuracy rates of over 85% for surgical instrument detection and classification. However, except for Bamba et al.'s results of Precision and Recall of about 98% and IoU of 0.77^21^, previous studies only have accuracy in the range of 38–86% and positive predictive value of 63–86% for instrument position detection^15^. Since the Dice of small objects tend to be small^22,23^ the accuracy of our study in identifying the approximately 1 mm-long HARMONIC tip was highly satisfactory.

In training environment studies, experts had shorter task execution times, shorter distances traveled by surgical instruments, lower velocity and acceleration peaks^11^, slower velocity^24^, and smoother movements^25^ compared to novices. However, it is not clear these differences would exist in a true clinical setting. In the present study, there were no differences in velocity, acceleration, or jerking between experts and novices. However, there was a tendency among novices for longer periods of slow movement where distance traveled per second was less than 120 pixels. Slow movements are the sum of “dwell time” and activation time. “Dwell time”^26^ indicates instrument hovering due to confusion. These results suggest that while training box tasks are easy to understand, novices may have periods of confusion during clinical surgery. Another point to consider is ideally the HARMONIC tip should remain stationary during activation. The tendency for experts to use it frequently with short pitches may also have affected kinematic evaluation results.

This study focused on fluctuation in motion analysis, and 1/f fluctuations can be seen from the cellular to the behavioral level^27^. The fluctuation index β of expert surgeons is higher and closer to 2 than novices, which indicates that expert surgical instrument movements are more regular. This is consistent with the training box results of Uemura et al.^28^ and informal impressions of surgeons. We believe that regular movement means “Confident, efficient, and safe conduct” which is the stated GOALS assessment criterion^6^, and the fluctuation index β quantifiably measures this. The correlation between β and the number of frames was Spearman's coefficient rho = -0.616, which was a moderate negative correlation. The number of frames – that is, the total surgical time – is an index that cannot be known until the end of the surgery, but β-value is an index that can be calculated in real time so skill evaluation can be performed during surgery. It is an index that evaluates movements over the past 3 s, and it may be possible to develop a system for real-time display on a surgical monitor. This will allow perioperative modifications if the β-value is below the 1.4 cutoff.

As a study limitation, it is important to note that results here are preliminary. Until now, task time^29^ and kinematic indices^30,31^ have been used as AI-based skill evaluation indices in dry box studies. Clinical surgery has seen a wide range of methods such as surgical time prediction based on procedure evaluation^32^, process prediction with heat maps^33^, and scoring of surgical field development^34^. As mentioned above, there are many AI-based skill evaluation indices, we have adopted kinematic indices as our evaluation indices. Although we have confidence results here show object detection technology can identify accurate positions on the screen, and kinematic indices can be obtained without using sensors, further research is necessary to identify the AI-based “gold standard”. This includes the need for more precise power analyses in future research, and more data from institutions and practitioners. Until now, only a few studies have used AI to identify coordinates of the instrument tip position to evaluate surgeon skill, and these studies are limited to heat-map assessments of tip position and total travel distance^16,17^. These studies have shown that experts move surgical instruments in a narrower field, but detailed studies of kinematic differences by skill level do not yet exist. This study is novel as it reveals that experts display efficient and regular motion of surgical instruments, and this can be evaluated in real time.

This study has other limitations in that it analyzed the position of surgical instruments from video, not their absolute position. Even if surgical instruments move the same distance, the distance moved appeared relatively small when the camera was far away and relatively large in close-up. Videos were 2D images, so depth direction movement was not captured. Also, the laparoscopic camera was held by an assistant, meaning movement and distance were not constant. As a result, data was not as reliable as sensor evaluation in an experimental environment.

This study focused solely on right-hand movements of surgeons, making it possible to evaluate them isolated from the support of skilled assistants. However, surgeon right-hand movements are only a small component of surgery. Although it is not clearly demarcated by the Endoscopic Surgical Skill Qualification System, the surgeon's right-hand accounts for only 19 points on the 60-point Common Criteria Scoring Table. In GOALS, it concerns only three of the five global scales. Further assessment items, such as left-hand movement and surgical field deployment, should be added to this study as well.

Furthermore, only six surgeons participated at a single institution and only 18 surgeries were evaluated. As mentioned earlier, in post hoc analysis fluctuation β displayed a high level of power at 0.81. That being said, in future data from more institutions and more surgeons is required. The expert surgeons in this study had performed more than 100 laparoscopic gastrectomies but evaluating top surgeons (1000 + cases) with this methodology could yield interesting results.

Overall, we believe clarifying differences between expert and novice surgeons is significant. In future, we would like to validate the methods in a multicenter setting to establish sufficient power, while expanding the evaluation method to include left-hand instrument movement, tissue position, and deployment evaluation.

## Conclusion

This article reported an original surgical skill evaluation method using artificial intelligence-based instrument identification. In a fixed procedure under actual clinical conditions, expert and novice surgeon groups did not show differences in right-hand ultrasonic coagulation shear tip movements of velocity, acceleration, or jerk. However, expert movements were more regular and total travel distance was shorter. Novice movements were comparatively irregular, with the shear tip tending towards longer hovering possibly due to confusion. Initial results here show promise for this evaluative protocol that can be validated further through large-scale multicenter studies.

### Supplementary Information

Supplementary Video 1.

Supplementary Information 1.

Supplementary Information 2.

Supplementary Information 3.

## Data Availability

All the data obtained and/or analyzed during the current study are available from the corresponding authors on reasonable request.
