# Supplementary material for: Laparoscopic distal gastrectomy skill evaluation from video: a new artificial intelligence-based instrument identification system
Source: Sci Rep. 2024 May 30;14:12432. doi: 10.1038/s41598-024-63388-y (PMC11139867; doi:10.1038/s41598-024-63388-y)
Supplement: Supplementary file 4 — Supplementary Information 3. [file 41598_2024_63388_MOESM4_ESM.docx]

Supplementary Material

Online Resource 1

AI analysis of the surgical video

The active blade was colored blue, the tissue pad was colored green, and the tip of the active blade was surrounded by a purple circle and colored to leave an afterimage of the previous 10 frames. The X and Y coordinates, velocity, acceleration, and jerk of the tip are displayed at the top left of the screen.

Online Resource 2,3

Part of the data and the code for calculating slope β
